# A Rare Complication of Ileus Following Endoscopic Ultrasound-Guided Celiac Plexus Neurolysis: A Case Report

**DOI:** 10.7759/cureus.10963

**Published:** 2020-10-15

**Authors:** Jack Sample, Faris Hammad, Sami Ghazaleh, Jordan Burlen, Ali Nawras

**Affiliations:** 1 Internal Medicine, University of Toledo, Toledo, USA; 2 Internal Medicine, Saint Vincent Charity Medical Center, Cleveland, USA; 3 Gastroenterology and Hepatology, University of Toledo, Toledo, USA

**Keywords:** ileus, endoscopic ultrasound, celiac plexus neurolysis, pancreatic cancer, palliative care

## Abstract

Pancreatic cancer patients experience debilitating pain, which makes pain management an integral part of the treatment plan. Endoscopic ultrasound-guided celiac plexus neurolysis (EUS-guided CPN) is an alternative palliative therapy for patients with pain due to pancreatic cancer. We report a patient who developed paralytic ileus after undergoing EUS-guided CPN.

A 77-year-old male patient presented with nausea, vomiting, and abdominal pain of one-day duration. He was diagnosed with stage IV pancreatic adenocarcinoma three weeks prior to presentation for which he underwent EUS-guided CPN. He had a 30-pack-year smoking history and quit 31 years ago. He reported moderate alcohol use and denied illicit drug use. In the emergency department, vital signs demonstrated normal blood pressure, heart rate, respiratory rate, and temperature. Abdominal exam was significant for minimal epigastric tenderness without guarding or rebound. Complete blood count (CBC), basic metabolic panel (BMP), and liver function tests were unremarkable. Computed tomography (CT) scan of the abdomen showed significant gastric distension. Esophagogastroduodenoscopy (EGD) showed large amounts of fluids within the gastric fundus and body. Upper gastrointestinal series showed delayed gastric emptying of the contrast, but contrast was seen in the third part of the duodenum and jejunum. Push enteroscopy showed no obstruction or mucosal abnormalities in the third or fourth parts of the duodenum. Small bowel obstruction was ruled out, and the diagnosis of ileus was made. The patient received ondansetron and polyethylene glycol as needed, and his diet was advanced slowly. His symptoms improved over the course of a few days, and he experienced a return of normal bowel activity. He eventually tolerated a regular diet and was discharged home in a stable condition.

Although EUS-guided CPN is a safe procedure, the procedure resulted in an unexpected ileus that has rarely been reported in the literature. Future studies with large sample sizes are recommended to capture the occurrence of the rare side effects of EUS-guided CPN.

## Introduction

Pancreatic cancer is the third leading cause of cancer-related deaths in the United States despite being the 11th most common cancer [1]. The incidence of pancreatic cancer deaths is similar to the number of newly diagnosed cases, and the National Cancer Institute predicts the incidence will increase to include 355,317 new cases by the year 2040 [2]. Pancreatic cancer is usually diagnosed at an advanced stage and lacks effective treatment options. Therefore, newly diagnosed pancreatic cancer is frequently managed by palliative care. 

Pancreatic cancer is notoriously associated with debilitating pain [3]. In advanced-stage pancreatic cancer, the primary goal of treatment is to improve quality of life. Optimizing pain management is difficult, and traditional analgesics are often unsuccessful. Additionally, high-dose opioid analgesic use may be detrimental to this population [4]. An alternative approach to pain management is necessary to ensure optimal palliation. Endoscopic ultrasound-guided celiac plexus neurolysis (EUS-guided CPN) is an effective alternative to pain management in pancreatic cancer. The use of EUS-guided CPN in pancreatic cancer has been shown to have excellent pain control in 89% of patients with 90% reporting partial or complete reduction of pain at three months [5]. This procedure is relatively safe but is not without complications. Common adverse effects are minor and include postprocedural pain, diarrhea, and asymptomatic hypotension secondary to unopposed parasympathetic activity [6]. Rarely, complications of this procedure can be severe. We present a case of paralytic ileus following EUS-guided CPN.

## Case presentation

A 77-year-old male patient presented to the emergency department with intractable nausea and bilious and non-bloody vomiting of one-day duration. This was associated with diffuse abdominal pain which he described as dull, non-radiating, and moderate in severity. His symptoms were exacerbated by oral intake. Prior to his presentation, he had normal bowel movements. He denied chest pain, shortness of breath, cough, dysphagia, diarrhea, or constipation. Past medical and surgical histories were noncontributory. The patient previously smoked 1.5 packs per day for 20 years but quit 31 years ago. He reported moderate alcohol use and denied illicit drug use.

Three weeks prior to his presentation to the emergency department, he was diagnosed with stage IV pancreatic adenocarcinoma. He did not receive chemotherapy and elected for conservative management as well as palliative care. One day prior to his presentation, he underwent EUS-guided CPN for palliation of his debilitating pain. The procedure involved injecting bupivacaine and 98% absolute alcohol under general anesthesia to induce unilateral celiac plexus neurolysis. There were no immediate post-procedure complications, and the patient was discharged home in a stable condition soon after completion of the neurolysis. Of note, the patient was using oxycodone 5 mg oral tablets three times per day as needed for pain since his cancer diagnosis. In the last three weeks prior to the procedure, he did not complain of nausea or constipation. He also denied taking the medication more frequently than usual.

In the emergency department, vital signs demonstrated blood pressure of 115/73 mmHg, heart rate of 82 beats per minute, respiratory rate of 15 breaths per minute, and temperature of 37.2℃. Abdominal exam was significant for minimal epigastric tenderness without guarding or rebound tenderness. The remainder of the physical exam was noncontributory. Complete blood count (CBC) showed white blood cell (WBC) of 10.0 × 103/µL, hemoglobin of 12.3 g/dL, and platelet count of 295 × 103/µL. Basic metabolic panel (BMP) showed sodium of 135 mmol/L, potassium of 5.1 mmol/L, chloride of 96 mmol/L, creatinine of 1.34 mg/dL, glucose of 384 mg/dL, and anion gap of 9 mmol/L. Liver function tests showed aspartate transaminase (AST) of 12 U/L, alanine transaminase (ALT) of 17 U/L, alkaline phosphatase of 74 U/L, and total bilirubin of 0.4 mg/dL. Lipase was within reference range at 5.0 U/L. Urinalysis did not reveal ketones. Hemoglobin A1c was unremarkable at 5.4% and glucose levels normalized without treatment, suggesting that the initial hyperglycemia was probably related to stress.

**Figure 1:**
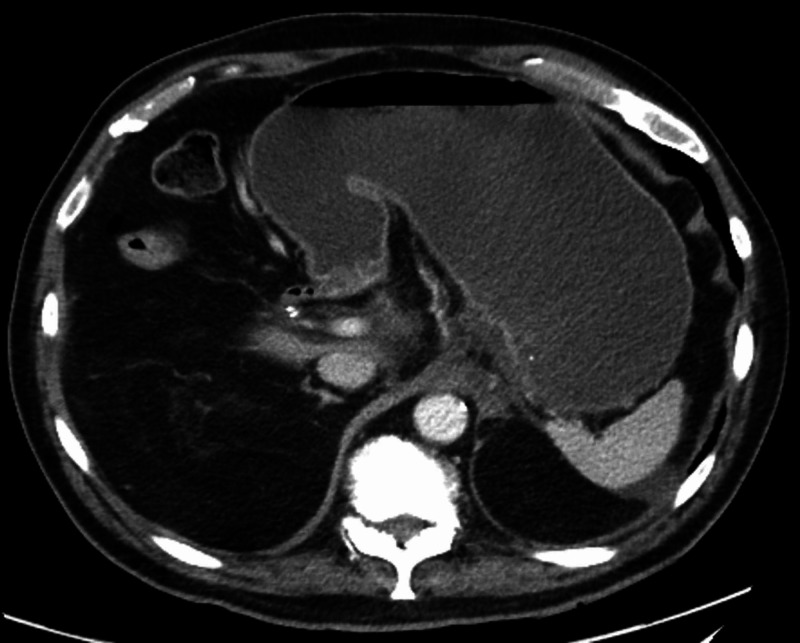
Computed tomography scan of the abdomen showing gastric distension.

The patient initially received intravenous fluids and was placed on a bowel rest regimen. Computed tomography (CT) scan of the abdomen showed significant gastric distension, which was concerning for obstruction at the level of the duodenum (Figure 1).

**Figure 2:**
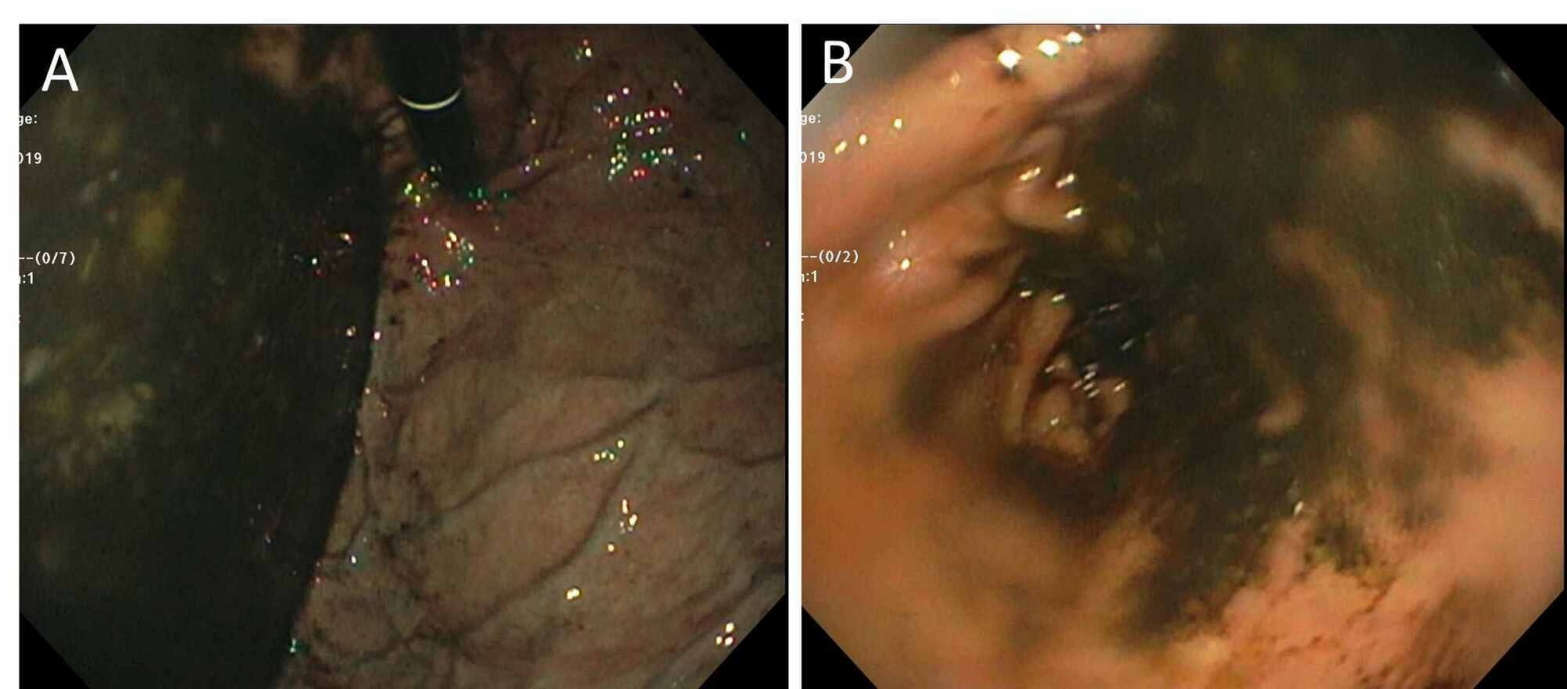
Esophagogastroduodenoscopy (EGD) showing large amounts of fluids within (A) gastric fundus and (B) gastric body.

Next, esophagogastroduodenoscopy (EGD) showed large amounts of fluids within the gastric fundus and body, which was also concerning for duodenal obstruction. The examined first and second parts of the duodenum were normal (Figure 2).

**Figure 3:**
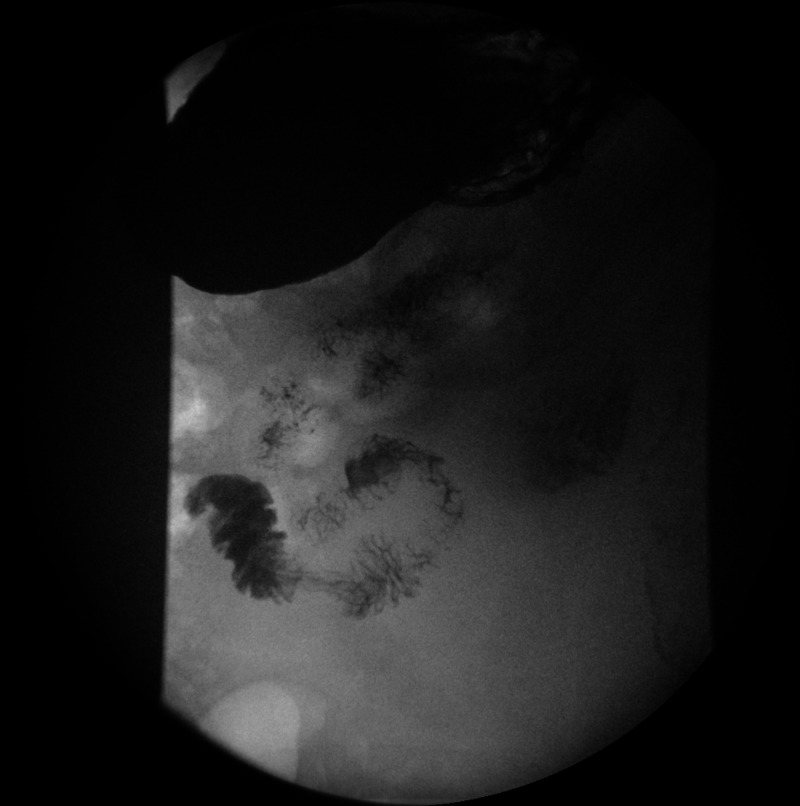
Upper gastrointestinal series showing contrast in the third part of the duodenum and jejunum.

As a result, upper gastrointestinal series with barium swallow was performed to look for an obstruction distal to the second part of the duodenum. Although it showed delayed gastric emptying of the contrast, contrast was seen in the third part of the duodenum and jejunum ruling out a complete obstruction in the duodenum (Figure 3).

**Figure 4:**
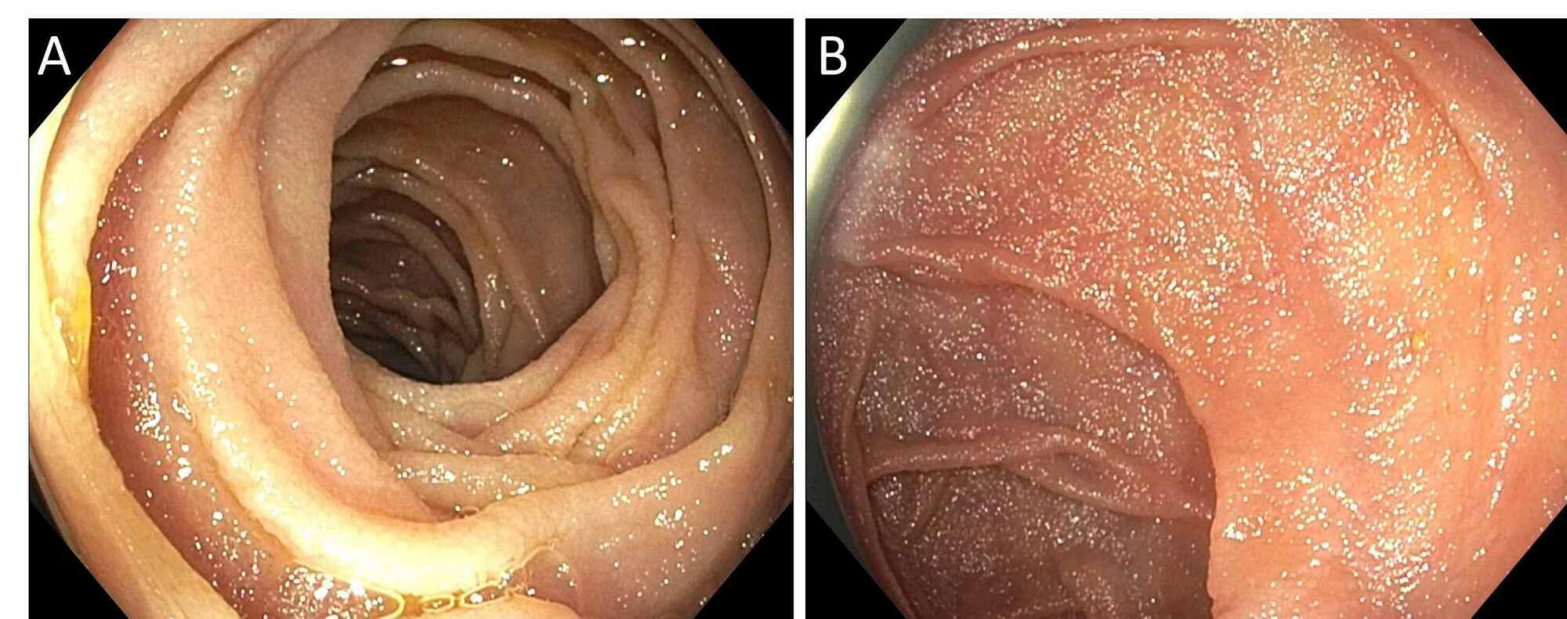
Push enteroscopy showing no obstruction or mucosal abnormalities in the (A) third or (B) fourth parts of the duodenum.

Finally, a push enteroscopy was performed to visualize the entire duodenum. It showed angulation in the third part of the duodenum but no obstruction or mucosal abnormalities in the third or fourth parts of the duodenum (Figure 4).

At that point, extensive gastrointestinal workup ruled out small bowel obstruction, and the diagnosis of ileus was made. The patient received ondansetron and polyethylene glycol as needed, and his diet was advanced slowly. His symptoms improved over the course of a few days, and he experienced a return of normal bowel activity. He eventually tolerated a regular diet and was discharged home in a stable condition. 

## Discussion

Many modalities can be employed in the management of pain in pancreatic adenocarcinoma. Traditionally, the mainstay approach was pharmacotherapy, including non-steroidal anti-inflammatory agents (NSAIDs) and opioid analgesics. However, these medications are often inadequate in their ability to reduce pain associated with pancreatic cancer and commonly cause significant undesirable side effects [7]. For these reasons, safe and effective alternative therapies are in high demand. These alternative therapies include CPN, celiac plexus block (CPB), splanchnicectomy, and intrathecal therapy [8-10].

CPN involves the interruption of the celiac plexus via chemical ablation with phenol or alcohol. Some studies even recommended its use as a first-line therapy, given its superior efficacy over traditional narcotics [11]. CPN can be performed under radiologic guidance using CT guidance or EUS. CT-guided CPN has higher rates of major complications including retroperitoneal bleeding, abscess, and neurovascular injuries [12]. Utilizing endoscopic ultrasound guidance provides better visualization of internal structures, which results in lower complication rates. Some studies also reported a favorable mortality outcome compared to other approaches [11].

CPN can cause unopposed parasympathetic activity, which may result in transient diarrhea and hypotension [12]. As a result, CPN is absolutely contraindicated in patients with existing intestinal obstruction or intraperitoneal infections. Our patient developed a clear presentation of paralytic ileus a day following CPN, which is unexpected following this procedure. Abdominal CT scans, EGD, upper gastrointestinal series with barium swallow, and push enteroscopy ruled out mechanical obstruction and favored a diagnosis of ileus. The exact mechanism by which EUS-guided CPN caused ileus in our patient is unknown. In our literature review, we found one other reported case of paralytic ileus following EUS-guided CPN [13]. Notably, that patient had pre-existing carcinomatous peritonitis with ascites, making the determination of direct etiology unclear.

## Conclusions

EUS-guided CPN is an alternative therapy for the optimization of pain management in pancreatic adenocarcinoma. This procedure has repeatedly been shown to be safe and effective; however, it may still cause unfavorable side effects. Commonly reported side effects include transient diarrhea and hypotension. In our case, the procedure unexpectedly resulted in ileus, which has rarely been reported in the current literature. Our patient improved with traditional conservative therapy, which is usually used to treat other forms of ileus including bowel rest, anti-emetics, and anti-laxatives. Providers should be aware of this rare adverse effect in patients who undergo EUS-guided CPN. Studies with large sample sizes are recommended to capture the occurrence of the rare side effects of EUS-guided CPN.
